# Comparative Efficacy and Safety of Metformin, Glyburide, and Insulin in Treating Gestational Diabetes Mellitus: A Meta-Analysis

**DOI:** 10.1155/2019/9804708

**Published:** 2019-11-04

**Authors:** Lanlan Guo, Jing Ma, Jia Tang, Dingyao Hu, Wei Zhang, Xue Zhao

**Affiliations:** ^1^Department of Radiation Oncology, Sun Yat-sen University Cancer Center, State Key Laboratory of Oncology in South China, Collaborative Innovation Center for Cancer Medicine, Guangzhou 510060, China; ^2^Department of Endocrinology and Metabolism, Gansu Provincial Hospital, Lanzhou 730000, China; ^3^Department of Infectious Diseases, Huashan Hospital, Fudan University, Shanghai 200041, China; ^4^The Second Clinical Medical College, Lanzhou University, Lanzhou 730000, China; ^5^Department of Critical Care Medicine, Affiliated Hospital of Zunyi Medical College, Zunyi 563000, China; ^6^Department of Nephrology, Shandong Provincial Hospital Affiliated to Shandong University, Jinan 250021, China

## Abstract

To compare the efficacy and safety of metformin, glyburide, and insulin in treating gestational diabetes mellitus (GDM), a meta-analysis of randomized controlled trials (RCTs) was conducted. PubMed, Embase, CINAHL, Web of Science, and Cochrane Library to November 13, 2018, were searched for RCT adjusted estimates of the efficacy and safety of metformin, glyburide, and insulin treatments in GDM patients. There were 41 studies involving 7703 GDM patients which were included in this meta-analysis; 12 primary outcomes and 24 secondary outcomes were detected and analyzed. Compared with metformin, insulin had a significant increase in the risk of preeclampsia (RR, 0.57; 95% CI, 0.45 to 0.72; *P* < 0.001), NICU admission (RR, 0.75; 95% CI, 0.64 to 0.87; *P* < 0.001), neonatal hypoglycemia (RR, 0.57; 95% CI, 0.49 to 0.66; *P* < 0.001), and macrosomia (RR, 0.68; 95% CI, 0.55 to 0.86; *P* < 0.05). To the outcomes of birth weight and gestational age at delivery, insulin had a significant increase when compared with metformin (MD, 114.48; 95% CI, 37.32 to 191.64; *P* < 0.01; MD, 0.23; 95% CI, 0.12 to 0.34; *P* < 0.001; respectively). Of the two groups between glyburide and metformin, metformin had lower gestational weight gain compared with glyburide (MD, 1.67; 95% CI, 0.26 to 3.07; *P* < 0.05). Glyburide had a higher risk of neonatal hypoglycemia compared with insulin (RR, 1.76; 95% CI, 1.32 to 2.36; *P* < 0.001). This meta-analysis found that metformin could be a safe and effective treatment for GDM. However, clinicians should pay attention on the long-term offspring outcomes of the relative data with GDM patients treated with metformin. Compared with insulin, glyburide had a higher increase of neonatal hypoglycemia. The use of glyburide in pregnancy for GDM women appears to be unclear.

## 1. Introduction

Gestational diabetes mellitus (GDM) is the most frequent medical complication of pregnancy and becoming a major global public health issue with the increasing prevalence in recent years due to the epidemic of obesity and type 2 diabetes. GDM affects about 7% of pregnancies in North America and has a global prevalence range from 5.8% to 12.9% and is associated with several maternal and neonatal adverse outcomes [1]. The presence of GDM always accompanies an increased maternal risk for preeclampsia and cesarean section and with an increased risk for developing type 2 diabetes (T2D) after pregnancy [2]. Moreover, GDM increases the risk of macrosomia, large for gestational age, shoulder dystocia, birth injury, neonatal hypoglycemia, preterm birth, hyperbilirubinemia, and others [3, 4]. Treatment of GDM can prevent short-term maternal and neonatal complications. The initial management for GDM includes nutritional modification and physical activity [5]. Almost 30% of women with GDM cannot be managed with diet and lifestyle modification alone and require pharmacological therapy to reduce the associated maternal and neonatal short- and long-term effects of GDM [6, 7].

Insulin historically has been considered the standard therapy for GDM management in cases refractory to nutrition therapy and exercise [7, 8], and this has continued to be reinforced by the ADA [8]. Insulin, which does not cross the placenta, lowers blood glucose by stimulating peripheral glucose uptake and inhibiting glucose production release by the liver [9]. However, it requires multiple daily injections and subsequently the need to train the patients in the technical aspect of treatment, resulting to more weight gain and higher medical cost [10–15]. In addition, hypoglycemia occurs in approximately 70% of women who use insulin some time during their pregnancy [16].

Oral antihyperglycemic drugs (OADs) (such as metformin and glyburide) can cross the placenta to the fetus. In addition, all oral agents lack long-term safety data. Therefore, they have not been approved by the U.S. Food and Drug Administration [17] and insulin continues to be the ADA recommended first-line therapy [8].

Metformin, as the first-line medication for T2DM, can promote glucose level control and lose weight and improve peripheral insulin resistance. Metformin is also known to increase the secretion of glucagon-like peptide 1 (GLP-1) from intestinal cells [18]. It is increasingly recognized as an alternative to insulin therapy for GDM [19, 20]. However, metformin has been found to have a maternal to fetal transfer and the long-term influence is uncertain. The largest study of metformin was in the MiG (metformin use in GDM) trial; Rowan et al. found that the primary composite outcome of neonatal morbidity was similar in the metformin arm compared to the insulin arm. Moreover, severe neonatal hypoglycemia was lower compared to women on insulin alone [21, 22].

Glyburide can stimulate the release of insulin from the pancreas. According to the recent study, Song et al. [23] reported that no significant differences in maternal short-term outcomes were observed between glyburide and insulin groups. Glyburide is a second-generation SU that can be considered safe and effective for the treatment of GDM. However, there are some concerns regarding a higher risk of macrosomia, large-for-gestational age infants, and neonatal hypoglycemia compared to insulin. Data regarding its use in GDM are conflicting in several studies [16, 23–29].

Because there is the paucity of adequate safety data, the use of these two drugs in GDM is restricted to the USA, although they are increasingly used now in Europe and South Africa [30]. In the past, oral hypoglycemic agents including metformin and glyburide have been used as alternative pharmacological treatment to insulin therapy [21, 31]. Nonetheless, the most recent 2019 American Diabetes Association guidelines do not recommend metformin and glyburide as first-line treatment for GDM, because they are known to cross the placenta and data on safety for offspring is lacking [32, 33]. In addition, the 2014 German Diabetes Association and German Association of Gynecology and Obstetrics guidelines do not recommend the use of oral hypoglycemic agents in GDM [34].

In recent years, several meta-analyses sought to assess the efficacy and safety of the treatment in GDM patients. In 2014, Jiang et al.'s study [14] including 18 RCTs revealed that both metformin and glyburide are suitable for use in the management of GDM, but glyburide was associated with more adverse pregnancy outcomes, including neonatal hypoglycemia, high maternal weight gain, high neonatal birth weight, and macrosomia. In 2017, Liang et al.'s study [28] including 31 RCTs revealed that metformin had more favorable pregnancy outcomes and the fastest rate of glucose control, especially in obese GDM patients, but with the lowest rate of average glucose control; glyburide have the highest rate of average glucose control, particularly in nonobese GDM patients, but with more adverse outcomes. The efficacy and safety of insulin, metformin, and glyburide in the treatment of GDM remain to be debated. Therefore, we performed a meta-analysis with the updated data, which might provide more evidence with respect to the efficacy and safety of metformin, glyburide, and insulin.

## 2. Materials and Methods

### 2.1. Ethic Statement

The protocol of this systematic review was registered in PROSPERO database on 8 March 2019 (CRD42019122611). This study was conducted according to the *Cochrane Handbook for Systematic Reviews of Interventions* [35], and the results were presented according to the PRISMA statement [36].

### 2.2. Search Strategy

A comprehensive electronic search strategy was conducted in PubMed, Embase, Web of Science, CINAHL, and Cochrane Library up to November 13, 2018. Authors of potentially eligible studies were contacted when necessary to request further information regarding study design or primary outcomes. The search strategies are included in Table 1.

### 2.3. Selection Criteria

Studies were included if they met the following criteria: subjects were women with gestational diabetes requiring drug treatment; the study was a randomized controlled trial that compares efficacy and safety parameters of metformin, glyburide, or insulin; the study provided information on one or more maternal or fetal outcome; they were published as a full paper. The exclusion criteria were as follows: reviews, letters, conferences abstract, case reports or series, comments, and animal experiment. Studies involving pregnant women with preexisting diabetes and studies with duplicated data were excluded.

### 2.4. Data Extraction and Quality Assessment

Two review authors independently assessed the quality of each included study by using the tool in the *Cochrane Handbook for Systematic Reviews of Intervention*. Two reviewers (Guo and Ma) independently performed the literature search, study selection, and data extraction. Differences in opinions were resolved by consensus with a third reviewer (Tang). When necessary, we contacted authors of original studies for additional data.

### 2.5. Outcomes of Interest

Outcomes of interest were divided into 2 categories: neonatal outcomes and maternal outcomes. There are 18 neonatal outcomes, including neonatal intensive care unit (NICU) admission, neonatal hypoglycemia (<2.2 mmol/L), macrosomia (>4 kg), sepsis, and respiratory distress syndrome (RDS). There are 17 maternal outcomes, including preeclampsia (blood pressure > 140/90 mmHg with proteinuria > 0.3 g/24 h), gestational hypertension, mode of delivery, maternal hypoglycemia (<3.3 mmol/L), pregnancy-induced hypertension (PIH), gestational weight gain, and HbA1c%.

### 2.6. Statistical Analysis

All analyses were performed using Review Manager 5.3 (Nordic Cochrane Centre). A fixed-effects model was used to pool the data if no significant heterogeneity was reported, and a random-effects model was used in the case of significant heterogeneity being used for an outcome, to calculate the risk ratio (RR) or mean difference (MD) and to assess the neonatal and maternal outcomes of different treatments in GDM patients. For continuous outcomes, we calculated mean differences (MD) and 95% confidence intervals (CI). For dichotomous outcomes, we calculated risk ratio (RR) and 95% CI. The heterogeneity was evaluated statistically by the chi-squared test (*P* < 0.1, *I*^2^ < 50%) and graphically using a forest or funnel plot analysis. If *I*^2^ > 50%, a random-effects model was used for the meta-analysis; if *I*^2^ < 50%, a fixed-effects model was used for the meta-analysis. *P* < 0.05 is considered to be statistically significant.

## 3. Results

### 3.1. Search Results

**Figure 1:**
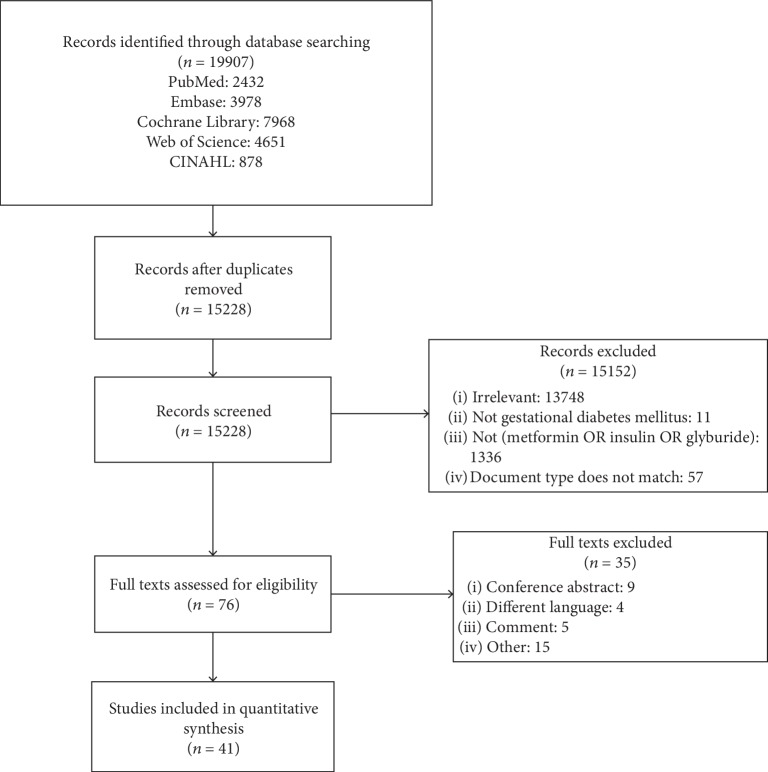
The search flow diagram.

The search retrieved 19907 abstracts. There were 15225 studies after duplicates were removed. Eventually, 41 studies fulfilled our inclusion criteria—23 comparing metformin with insulin (4674 subjects), 13 comparing glibenclamide with insulin (2561subjects), and 5 comparing metformin with glibenclamide (684 subjects) [21, 24, 25, 29, 31, 37–72].  Figure 1 shows the search flow diagram. The characteristics of the included studies are described in Table 2.

### 3.2. Assessment of Risk of Bias

**Figure 2:**
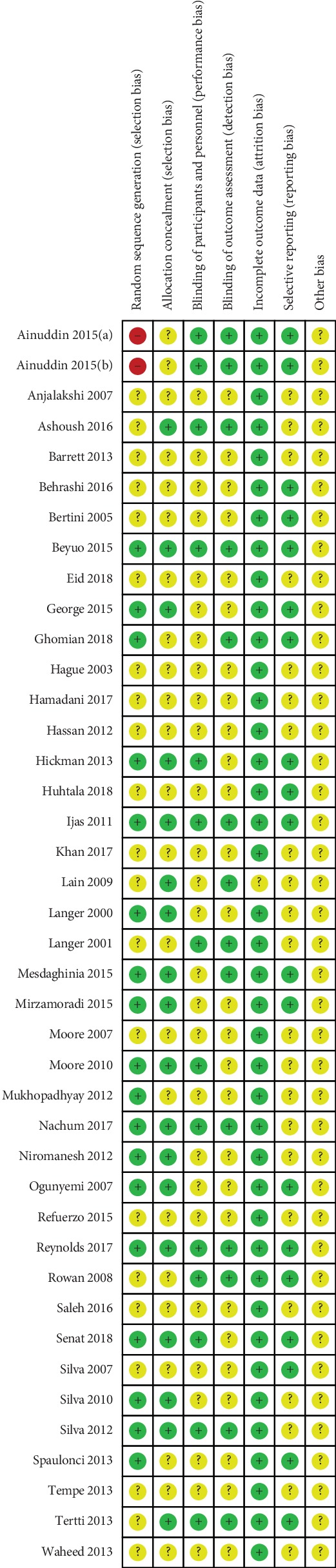
Risk of bias summary.

**Figure 3:**
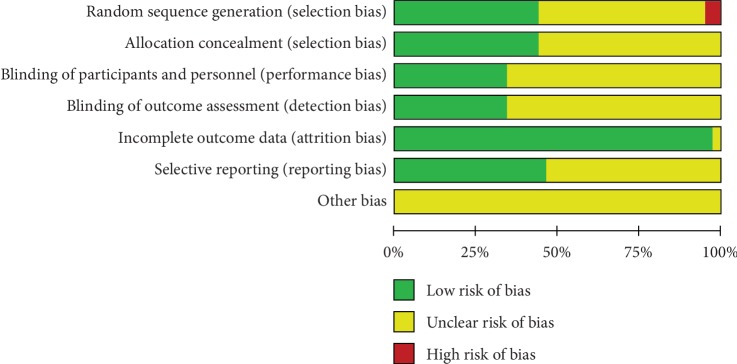
Risk of bias graph.

Due to the high risk of bias, the results from the aforementioned studies were analyzed separately as required to determine whether the conclusions were affected by the inclusion of these “high-risk” studies. Two review authors independently assessed the risk of bias for each included study by using the Cochrane Collaboration's risk-of-bias tool in the *Cochrane Handbook for Systematic Reviews of Interventions* [73]. The quality assessments of the included studies are presented in Figures 2 and 3.

### 3.3. Effects of Intervention

In the forest and funnel plots, studies were categorized into the following groups: “metformin vs. insulin,” “glyburide vs. insulin,” and “glyburide vs. metformin.” The complete set of forest plots and funnel plots are available in the appendix.

#### 3.3.1. Preterm Birth

Preterm birth was included as an outcome by 11 studies which involved 2943 GDM patients. There was significant heterogeneity between these studies (*P* < 0.001, *I*^2^ = 71%). The pooled result showed no significant statistical difference between the metformin and insulin groups in terms of preterm birth (RR, 0.90; 95% CI, 0.51 to 1.58; *P* = 0.71). Preterm birth was reported as an outcome between glyburide and insulin by 3 studies which included 941 GDM patients. There was no significant heterogeneity between these studies (*P* = 0.81, *I*^2^ = 0%). There was no significant statistical difference between the glyburide and insulin groups in terms of preterm birth (RR, 1.58; 95% CI, 0.90 to 2.76; *P* = 0.11).

#### 3.3.2. Hypertensive Disorders

*(1) Gestational Hypertension*. Gestational hypertension was included as an outcome by 5 studies which involved 1388 GDM patients. There was no significant heterogeneity between 5 studies (*P* = 0.67, *I*^2^ = 0%). In the pairwise meta-analysis, we observed that metformin had lower incidence of preeclampsia compared with insulin (RR, 0.56; 95% CI, 0.36 to 0.87; *P* < 0.01).

*(2) Pregnancy-Induced Hypertension (PIH)*. Pregnancy-induced hypertension was included as an outcome between metformin and insulin by 3 studies which involved 606 GDM patients. There was no significant heterogeneity between these studies (*P* = 0.52, *I*^2^ = 0%). Data showed no significant statistical difference between the metformin and insulin groups in terms of pregnancy-induced hypertension (RR, 0.56; 95% CI, 0.30 to 1.06; *P* = 0.08).

*(3) Preeclampsia*. Preeclampsia was included as an outcome between metformin and insulin by 14 studies which involved 3402 GDM patients. There was no significant heterogeneity between these studies (*P* = 0.05, *I*^2^ = 43%). In the pairwise meta-analysis, we observed that metformin had lower incidence of preeclampsia than insulin (RR, 0.57; 95% CI, 0.45 to 0.72; *P* < 0.001). Three studies involving 564 GDM patients focused on the incidence of preeclampsia between glyburide and insulin. There was no significant heterogeneity between these studies (*P* = 0.58, *I*^2^ = 0%). The pooled result showed no significant statistical difference between the glyburide and insulin groups in terms of preeclampsia (RR, 0.98; 95% CI, 0.56 to 1.74; *P* = 0.95).

#### 3.3.3. Mode of Delivery

*(1) Induction of Labor*. Induction of labor was included as an outcome by 8 studies which involved 1066 GDM patients. There was no significant heterogeneity between these studies (*P* = 0.10, *I*^2^ = 42%). In the pairwise meta-analysis, we observed that metformin was associated with a significantly reduced incidence of induction of labor compared with insulin (RR, 0.85; 95% CI, 0.74 to 0.99; *P* < 0.05).

*(2) Cesarean Section*. Cesarean section was included as an outcome between metformin and insulin by 15 studies which involved 2611 GDM patients. There was no significant heterogeneity between these studies (*P* = 0.12, *I*^2^ = 31%). There was no significant statistical difference between the metformin and insulin groups in terms of cesarean section (RR, 1.00; 95% CI, 0.90 to 1.10; *P* = 0.96). Five studies involving 1429 GDM patients reported the cesarean section between glyburide and insulin. There was no significant heterogeneity between these studies (*P* = 0.70, *I*^2^ = 0%). The pooled result showed no significant statistical difference between the glyburide and insulin groups in terms of cesarean section (MD, 0.89; 95% CI, 0.71 to 1.13; *P* = 0.35). Four studies involving 525 GDM patients focused on the incidence of cesarean section between glyburide and metformin. There was significant heterogeneity between these studies (*P* = 0.10, *I*^2^ = 52%). The pooled result showed no significant statistical difference between the glyburide and metformin groups in terms of cesarean delivery (RR, 0.95; 95% CI, 0.71 to 1.27; *P* = 0.73).

*(i) Elective Cesarean Section*. Elective cesarean section was included as an outcome between metformin and insulin by 3 studies which involved 526 GDM patients. There was no significant heterogeneity between these studies (*P* = 0.16, *I*^2^ = 46%). In the pairwise meta-analysis, we observed that metformin had lower incidence of elective cesarean section compared with insulin (RR, 0.73; 95% CI, 0.54 to 1.00; *P* = 0.05).

*(ii) Emergency Cesarean Section*. Emergency cesarean section was included as an outcome between metformin and insulin by 3 studies which involved 526 GDM patients. There was no significant heterogeneity between these studies (*P* = 0.32, *I*^2^ = 13%). However, there was no significant statistical difference between the metformin and insulin groups in terms of emergency cesarean section (RR, 1.10; 95% CI, 0.82 to 1.49; *P* = 0.52).

*(3) Vaginal Delivery*. Vaginal delivery was included as an outcome between metformin and insulin by 8 studies which involved 1206 GDM patients. There was no significant heterogeneity between these studies (*P* = 0.10, *I*^2^ = 42%). However, there was no significant statistical difference between the metformin and insulin groups in terms of vaginal delivery (RR, 1.12; 95% CI, 0.99 to 1.25; *P* = 0.06).

*(i) Assisted Vaginal Delivery*. Assisted vaginal delivery was included as an outcome between metformin and insulin by 4 studies which involved 667 GDM patients. There was no significant heterogeneity between these studies (*P* = 0.70, *I*^2^ = 0%). However, there was no significant statistical difference between the metformin and insulin groups in terms of assisted vaginal delivery (RR, 1.06; 95% CI, 0.63 to 1.80; *P* = 0.82).

*(ii) Spontaneous Vaginal Delivery*. Spontaneous vaginal delivery was included as an outcome between metformin and insulin by 4 studies which involved 680 GDM patients. There was significant heterogeneity between these studies (*P* = 0.04, *I*^2^ = 65%). The pooled result showed no significant statistical difference between the metformin and insulin groups in terms of spontaneous vaginal delivery (RR, 0.97; 95% CI, 0.54 to 1.74; *P* = 0.96).

#### 3.3.4. Maternal Hypoglycemia

Maternal hypoglycemia was included as an outcome between metformin and insulin by 3 studies which involved 352 GDM patients. There was no significant heterogeneity between these studies (*P* = 0.49, *I*^2^ = 0%). In the pairwise meta-analysis, we observed that metformin had lower incidence of maternal hypoglycemia compared with insulin (RR, 0.28; 95% CI, 0.10 to 0.75; *P* = 0.05).

#### 3.3.5. Gestational Age at Delivery

Cesarean delivery was included as an outcome between insulin and metformin by 12 studies which involved 2295 GDM patients. There was no significant heterogeneity between these studies (*P* = 0.55, *I*^2^ = 0%). In the pairwise meta-analysis, we observed that metformin had lower gestational age at delivery compared with insulin (MD, 0.23; 95% CI, 0.12 to 0.34; *P* < 0.001). Seven studies involving 1007 GDM patients reported the gestational age at delivery between glyburide and insulin. There was significant heterogeneity between these studies (*P* < 0.001, *I*^2^ = 79%). The pooled result showed no significant statistical difference between the glyburide and insulin groups in terms of gestational age at delivery (MD, 0.14; 95% CI, -0.32 to 0.61; *P* = 0.55). Four studies involving 535 GDM patients reported the gestational age at delivery between glyburide and metformin. There was no significant heterogeneity between these studies (*P* = 0.17, *I*^2^ = 40%). However, the pooled result showed no significant statistical difference between the glyburide and metformin groups in terms of gestational age at delivery (MD, 0.10; 95% CI, -0.13 to 0.33; *P* = 0.39).

#### 3.3.6. Gestational Weight Gain

Gestational weight gain was included as an outcome between insulin and metformin by 9 studies which involved 1135 GDM patients. In the pairwise meta-analysis, we observed that metformin had lower gestational weight gain compared with insulin (MD, 1.29; 95% CI, 0.40 to 2.19; *P* < 0.001). However, there was significant heterogeneity between these studies (*P* < 0.001, *I*^2^ = 84%). Gestational weight gain was reported as an outcome between insulin and glyburide by 3 studies which included 523 GDM patients. There was no significant heterogeneity between these studies (*P* = 0.41, *I*^2^ = 0%). However, there was no significant statistical difference between the insulin and glyburide groups in terms of gestational weight gain (MD, 0.66; 95% CI, -0.36 to 1.69; *P* = 0.20). Three studies involving 376 GDM patients reported the gestational weight gain between glyburide and metformin. There was no significant heterogeneity between these studies (*P* = 0.45, *I*^2^ = 0%). In the pairwise meta-analysis, we observed that metformin had lower gestational weight gain compared with glyburide (MD, 1.67; 95% CI, 0.26 to 3.07; *P* = 0.02).

#### 3.3.7. Glycemic Control (the HbA1c% at Delivery, Glycated Hemoglobin at Weeks 36-37)

The HbA1c% at delivery was included as an outcome between insulin and metformin by 5 studies which involved 932 GDM patients. There was no significant heterogeneity between these studies (*P* = 0.84, *I*^2^ = 0%). In the pairwise meta-analysis, we observed that metformin had the lower HbA1c% at weeks 36-37 compared with insulin (MD, 0.18; 95% CI, 0.07 to 0.29; *P* < 0.01).

Glycated hemoglobin at weeks 36-37 was reported as an outcome between metformin and insulin by 6 studies which involved 1539 GDM patients. There was significant heterogeneity between these studies (*P* < 0.0001, *I*^2^ = 84%). The pooled result showed no significant statistical difference between the metformin and insulin groups in terms of glycated hemoglobin at weeks 36-37 (MD, 0.06; 95% CI, -0.05 to 0.18; *P* = 0.29).

#### 3.3.8. Fasting Blood Glucose (FBG)

FBG was included as an outcome between metformin and insulin by 9 studies which involved 2641 GDM patients. There was significant heterogeneity between these studies (*P* < 0.00001, *I*^2^ = 95%). The pooled result showed no significant statistical difference between the metformin and insulin groups in terms of FBG (MD, 0.64; 95% CI, -1.56 to 2.84; *P* = 0.87). Three studies involving 582 GDM patients reported the FBG between insulin and glyburide. There was significant heterogeneity between these studies (*P* < 0.001, *I*^2^ = 86%). The pooled result showed no significant statistical difference between the insulin and glyburide in terms of FBG (MD, 2.54; 95% CI, -4.98 to 10.06; *P* = 0.51).

#### 3.3.9. Two-Hour Postprandial Glucose (2HPG)

2HPG was included as an outcome between insulin and metformin by 6 studies which involved 2315 GDM patients. There was significant heterogeneity between these studies (*P* < 0.001, *I*^2^ = 92%). The pooled result showed no significant statistical difference between the insulin and metformin groups in terms of 2HPG (MD, 1.61; 95% CI, -0.34 to 3.56; *P* = 0.11).

#### 3.3.10. NICU Admission

NICU admission was included as an outcome between metformin and insulin by 14 studies which involved 2402 GDM patients. There was no significant heterogeneity between these studies (*P* = 0.60, *I*^2^ = 0%). In the pairwise meta-analysis, we observed that metformin had lower incidence of NICU admission compared with insulin (RR, 0.75; 95% CI, 0.64 to 0.87; *P* < 0.001). NICU admission was reported as an outcome between glyburide and insulin by 7 studies which included 1751 GDM patients. There was no significant heterogeneity between these studies (*P* = 0.65, *I*^2^ = 0%). However, there was no significant statistical difference between the glyburide and insulin groups in terms of NICU admission (OR, 0.94; 95% CI, 0.58 to 1.51; *P* = 0.78). Three studies involving 421 GDM patients focused on the incidence of NICU admission between glyburide and metformin. There was no significant heterogeneity between these studies (*P* = 0.42, *I*^2^ = 0%). However, there was no significant statistical difference between the glyburide and metformin groups in terms of NICU admission (RR, 0.55; 95% CI, 0.26 to 1.16; *P* = 0.12).

#### 3.3.11. Need for Neonatal Dextrose

Need for neonatal dextrose was included as an outcome between metformin and insulin by 3 studies which involved 255 GDM patients. There was no significant heterogeneity between these studies (*P* = 0.45, *I*^2^ = 0%). However, there was no significant statistical difference between the metformin and insulin groups in terms of need for neonatal dextrose (RR, 1.06; 95% CI, 0.67 to 1.68; *P* = 0.81).

#### 3.3.12. Neonatal Hypocalcemia

Neonatal hypocalcemia was included as an outcome between glyburide and insulin by 3 studies which involved 749 GDM patients. There was no significant heterogeneity between these studies (*P* = 0.26, *I*^2^ = 20%). However, there was no significant statistical difference between the glyburide and insulin groups in terms of neonatal hypocalcemia (OR, 0.53; 95% CI, 0.11 to 2.63; *P* = 0.43).

#### 3.3.13. Congenital Anomaly

Congenital anomaly was included as an outcome between metformin and insulin by 6 studies which involved 839 GDM patients. There was no significant heterogeneity between these studies (*P* = 0.31, *I*^2^ = 17%). However, there was no significant statistical difference between the metformin and insulin groups in terms of congenital anomaly (OR, 0.78; 95% CI, 0.29 to 2.11; *P* = 0.63). Seven studies involving 1049 GDM patients focused on the incidence of the congenital anomaly between glyburide and insulin. There was no significant heterogeneity between these studies (*P* = 0.87, *I*^2^ = 0%). However, there was no significant statistical difference between the glyburide and insulin groups in terms of congenital anomaly (RR, 1.06; 95% CI, 0.73 to 1.54; *P* = 0.76).

#### 3.3.14. Neonatal Hypoglycemia

Neonatal hypoglycemia was included as an outcome between metformin and insulin by 15 studies which involved 2755 GDM patients. There was no significant heterogeneity between these studies (*P* = 0.66, *I*^2^ = 0%). In the pairwise meta-analysis, we observed that metformin had lower incidence of neonatal hypoglycemia compared with insulin (RR, 0.57; 95% CI, 0.49 to 0.66; *P* < 0.00001). Neonatal hypoglycemia was reported as an outcome between glyburide and insulin by 12 studies which included 2406 GDM patients. There was no significant heterogeneity between these studies (*P* = 0.21, *I*^2^ = 25%). In the pairwise meta-analysis, we observed that glyburide had higher incidence of neonatal hypoglycemia compared with insulin (RR, 1.76; 95% CI, 1.32 to 2.36; *P* < 0.001). Five studies involving 684 GDM patients focused on the incidence of neonatal hypoglycemia between glyburide and metformin. There was no significant heterogeneity between these studies (*P* = 0.09, *I*^2^ = 50%). However, there was no significant statistical difference between the glyburide and metformin groups in terms of neonatal hypoglycemia (RR, 1.03; 95% CI, 0.39 to 2.74; *P* = 0.95).

#### 3.3.15. Birth Injury

Birth injury was included as an outcome between metformin and insulin by 7 studies which involved 1769 GDM patients. There was significant heterogeneity between these studies (*P* = 0.11, *I*^2^ = 55%). Also, there was no significant statistical difference between the metformin and insulin groups in terms of birth injury (OR, 1.12; 95% CI, 0.66 to 1.89; *P* = 0.67).

#### 3.3.16. Sepsis

Sepsis was included as an outcome between metformin and insulin by 4 studies which involved 1167 GDM patients. There was significant heterogeneity between these studies (*P* = 0.11, *I*^2^ = 55%). Also, there was no significant statistical difference between the metformin and insulin groups in terms of birth injury (OR, 1.12; 95% CI, 0.66 to 1.89; *P* = 0.67).

#### 3.3.17. Five-Minute Apgar Score

The 5-minute Apgar score was included as an outcome between insulin and metformin and by 8 studies which involved 1059 GDM patients. There was significant heterogeneity between these studies (*P* < 0.001, *I*^2^ = 79%). The pooled result showed no significant statistical difference between the insulin and metformin groups in terms of the 5-minute Apgar score (RR, 0.05; 95% CI, -0.19 to 0.28; *P* = 0.68).

#### 3.3.18. Five-Minute Apgar Score < 7

The 5-minute Apgar score < 7 was included as an outcome between metformin and insulin by 5 studies which involved 1585 GDM patients. There was no significant heterogeneity between these studies (*P* = 0.88, *I*^2^ = 0%). However, there was no significant statistical difference between the metformin and insulin groups in terms of the 5-minute Apgar score < 7 (OR, 1.29; 95% CI, 0.70 to 2.38; *P* = 0.42). The 5-minute Apgar score < 7 was reported as an outcome between glyburide and metformin by 3 studies which included 325 GDM patients. There was no significant heterogeneity between these studies (*P* = 0.30, *I*^2^ = 7%). However, there was no significant statistical difference between the glyburide and metformin groups in terms of the 5-minute Apgar score < 7 (RR, 0.88; 95% CI, 0.13 to 5.89; *P* = 0.90).

#### 3.3.19. Macrosomia

Macrosomia was included as an outcome between metformin and insulin by 13 studies which involved 2331 GDM patients. There was no significant heterogeneity between these studies (*P* = 0.38, *I*^2^ = 6%). In the pairwise meta-analysis, we observed that metformin had lower incidence of macrosomia compared with insulin (RR, 0.68; 95% CI, 0.55 to 0.86; *P* < 0.05). Nine studies involving 2227 GDM patients focused on the incidence of macrosomia between glyburide and insulin. There was significant heterogeneity between these studies (*P* = 0.02, *I*^2^ = 56%). The pooled result showed no significant statistical difference in terms of macrosomia (RR, 1.46; 95% CI, 0.78 to 2.75; *P* = 0.24). Four studies involving 484 GDM patients focused on the incidence of macrosomia between glyburide and metformin. There was no significant heterogeneity between these studies (*P* = 0.39, *I*^2^ = 1%). However, there was no significant statistical difference between the glyburide and metformin groups in terms of macrosomia (OR, 1.45; 95% CI, 0.63 to 3.37; *P* = 0.39).

#### 3.3.20. Respiratory Distress Syndrome (RDS)

RDS was included as an outcome between metformin and insulin by 12 studies which involved 2172 GDM patients. There was significant heterogeneity between these studies (*P* = 0.01, *I*^2^ = 55%). Also, there was no significant statistical difference between the metformin and insulin groups in terms of RDS (OR, 1.03; 95% CI, 0.68 to 1.56; *P* = 0.88).

#### 3.3.21. Shoulder Dystocia

Shoulder dystocia was included as an outcome between metformin and insulin by 6 studies which involved 625 GDM patients. There was no significant heterogeneity between these studies (*P* = 0.15, *I*^2^ = 40%). However, there was no significant difference between the metformin and insulin groups in terms of shoulder dystocia (OR, 1.33; 95% CI, 0.36 to 4.94; *P* = 0.67).

#### 3.3.22. Neonatal Jaundice/Hyperbilirubinemia

Neonatal jaundice/hyperbilirubinemia was included as an outcome between metformin and insulin by 13 studies which involved 2378 GDM patients. There was no significant heterogeneity between these studies (*P* = 0.03, *I*^2^ = 47%). However, there was no significant statistical difference between the metformin and insulin groups in terms of neonatal jaundice/hyperbilirubinemia (RR, 1.07; 95% CI, 0.94 to 1.23; *P* = 0.31). Neonatal jaundice/hyperbilirubinemia was reported as an outcome between glyburide and insulin by 5 studies which included 1618 GDM patients. There was no significant heterogeneity between these studies (*P* = 0.43, *I*^2^ = 0%). However, there was no significant statistical difference between the glyburide and insulin groups in terms of neonatal jaundice/hyperbilirubinemia (RR, 1.09; 95% CI, 0.84 to 1.41; *P* = 0.52).

#### 3.3.23. Large for Gestational Age (>90th Percentile)

LGA (>90th percentile) was included as an outcome between metformin and insulin by 13 studies which involved 2812 GDM patients. There was no significant heterogeneity between these studies (*P* = 0.51, *I*^2^ = 0%). However, there was no significant statistical difference between the metformin and insulin groups in terms of LGA (RR, 0.87; 95% CI, 0.74 to 1.02; *P* = 0.09). Five studies involving 1006 GDM patients focused on the incidence of LGA between glyburide and insulin. There was significant heterogeneity between these studies (*P* = 0.02, *I*^2^ = 66%). The pooled result showed no significant statistical difference between the glyburide and insulin groups in terms of LGA (RR, 1.66; 95% CI, 0.83 to 3.31; *P* = 0.15). Four studies involving 376 GDM patients focused on the incidence of LGA between glyburide and metformin. There was significant heterogeneity between these studies (*P* = 0.03, *I*^2^ = 72%). The pooled result showed no significant statistical difference between the glyburide and metformin groups in terms of LGA (RR, 0.67; 95% CI, 0.25 to 1.76; *P* = 0.41).

#### 3.3.24. Small for Gestational Age (<10th Percentile)

SGA (<10th percentile) was included as an outcome between metformin and insulin by 12 studies which involved 2833 GDM patients. There was no significant heterogeneity between these studies (*P* = 0.04, *I*^2^ = 47%). However, there was no significant statistical difference between the metformin and insulin groups in terms of SGA (RR, 1.06; 95% CI, 0.82 to 1.37; *P* = 0.65).

#### 3.3.25. Transient Tachypnea

Transient tachypnea was included as an outcome between metformin and insulin by 4 studies which involved 1104 GDM patients. There was no significant heterogeneity between these studies (*P* = 0.92, *I*^2^ = 0%). However, there was no significant statistical difference between the metformin and insulin groups in terms of transient tachypnea (OR, 0.76; 95% CI, 0.36 to 1.57; *P* = 0.45).

#### 3.3.26. Birth Weight

Birth weight was included as an outcome between metformin and insulin by 16 studies which involved 2853 GDM patients. There was significant heterogeneity between these studies (*P* < 0.001, *I*^2^ = 83%). However, in the pairwise meta-analysis, we observed that metformin had lower birth weight compared with insulin (MD, 114.48; 95% CI, 37.32 to 191.64; *P* < 0.01). 10 studies involving 1980 GDM patients reported the birth weight between glyburide and insulin. There was significant heterogeneity between these studies (*P* < 0.001, *I*^2^ = 86%). The pooled result showed no significant statistical difference between the glyburide and insulin groups in terms of birth weight (MD, 62.58; 95% CI, -55.98 to 181.14; *P* = 0.30). Six studies involving 707 GDM patients reported the birth weight between glyburide and metformin. There was significant heterogeneity between these studies (*P* = 0.03, *I*^2^ = 61%). The pooled result showed no significant statistical difference between the glyburide and metformin groups in terms of birth weight (MD, 92.64; 95% CI, -10.60 to 195.88; *P* = 0.08).

#### 3.3.27. Umbilical Artery pH

Umbilical artery pH was included as an outcome between metformin and insulin by 6 studies which involved 961 GDM patients. There was no significant heterogeneity between these studies (*P* = 0.98, *I*^2^ = 0%). However, there was no significant statistical difference between the metformin and insulin groups in terms of umbilical artery pH (MD, 0.00; 95% CI, -0.01 to 0.01; *P* = 0.64).

#### 3.3.28. Neonatal Blood Glucose

Neonatal blood glucose was included as an outcome between metformin and insulin by 3 studies which involved 384 GDM patients. There was no significant heterogeneity between these studies (*P* = 0.17, *I*^2^ = 44%). In the pairwise meta-analysis, we observed that insulin had lower neonatal blood glucose compared with metformin (MD, 2.95; 95% CI, 0.63 to 5.26; *P* < 0.05).

## 4. Discussion

The purpose of this meta-analysis was to evaluate the efficacy and safety of three drugs (metformin, glyburide, and insulin) for GDM. Several maternal and neonatal outcomes were assessed.

Our meta-analysis, in accordance with the result of previous review [74], suggested that metformin could be a safe and effective treatment for GDM. There was no significant difference between metformin and insulin in terms of glycemic control; the other comparative two groups did not reveal a significant difference either. Differently [74], metformin had a lower risk of preeclampsia compared with insulin groups (RR, 0.57; 95% CI, 0.45 to 0.72; *P* < 0.001). To the outcomes of neonatal blood glucose and birth weight, metformin had a lower increase when compared with insulin (MD, 2.95; 95% CI, 0.63 to 5.26; *P* < 0.05; MD, 114.48; 95% CI, 37.32 to 191.64; *P* < 0.01; respectively). Mothers receiving metformin had a lower incidence of maternal hypoglycemia (RR, 0.28; 95% CI, 0.10 to 0.75; *P* = 0.05), gestational hypertension (RR, 0.56; 95% CI, 0.36 to 0.87; *P* < 0.01), and induction of labor (RR, 0.85; 95% CI, 0.74 to 0.99; *P* < 0.05) than those in the insulin group. Compared with metformin, insulin had a significant increase in the gestational age at delivery (MD, 0.23; 95% CI, 0.12 to 0.34; *P* < 0.001); likewise, insulin had a risk of macrosomia (RR, 0.68; 95% CI, 0.55 to 0.86; *P* < 0.05). Risks of NICU admission and neonatal hypoglycemia were lower in the metformin group and reached a statistically significant level when compared with insulin (RR, 0.75; 95% CI, 0.64 to 0.87; *P* < 0.001; RR, 0.57; 95% CI, 0.49 to 0.66; *P* < 0.001; respectively). We observed no statistically significant difference in other outcomes. However, the latest study [75] showed that with dietary and lifestyle advice started at 10-20 weeks' gestation when metformin was given to overweight or obese pregnant women, metformin cannot improve pregnancy and birth outcomes. Statistically significant heterogeneity prevented analyses of the outcomes of gestational weight gain and birth weight. The outcomes for other differences between the two groups remained nonsignificant.

Metformin had higher gestational weight gain compared with glyburide (MD, 1.67; 95% CI, 0.26 to 3.07; *P* < 0.05). For the rest of the outcomes, no significant difference was noticed between the two groups. Of the two groups between glyburide and insulin, glyburide had a higher risk of neonatal hypoglycemia compared with insulin (RR, 1.76; 95% CI, 1.32 to 2.36; *P* < 0.001), which is the same as the study [23]. In the study [22], significant differences for outcomes in between glyburide and insulin were obtained in birth weight (MD, 109; 95% CI, 35.9 to 181; *P* < 0.01), macrosomia (RR, 2.62; 95% CI, 1.35 to 5.08; *P* < 0.01), and neonatal hypoglycemia (RR, 2.04; 95% CI, 1.30 to 3.20; *P* < 0.01), which are different from our study, but in our meta-analysis, there are more subjects. No other significant difference was noticed between the two groups.

In the meta-analysis [28], glyburide ranked the worst with the highest incidence of macrosomia, preeclampsia, hyperbilirubinemia, neonatal hypoglycemia, preterm birth, and low birth weight; metformin (plus insulin when required) has the lowest risk of macrosomia, pregnancy hypertension, LGA, RDS, preterm birth, and low birth weight. Besides, insulin had the highest incidence of NICU admission.

Our findings, which are based on more recent studies, except for preterm birth, are in accordance with the results of previous meta-analyses [6, 16, 17, 23, 73, 75–81]. Gui et al.'s study [73] including 3 RCTs revealed that metformin had a significantly higher risk than insulin in terms of preterm birth, the incidence of preterm birth was significantly higher in the metformin group than in the insulin group (OR, 1.74; 95% CI, 1.13 to 2.68; *P* = 0.01); also, there was no significant heterogeneity between these studies (*P* = 0.84, *I*^2^ = 0%). In our meta-analysis, which has 11 RCTs, in addition, we calculated RR to analyze the data. Su and Wang's study [81] including 6 RCTs revealed that metformin had a higher incidence of preterm birth compared with insulin (RR, 1.56; 95% CI, 1.06 to 2.30; *P* = 0.01). In this meta-analysis, there is no forest plot about each evaluation index and the search flow diagram; besides, our meta-analysis has increased sample size. Therefore, our results are more reliable. In contrast to other meta-analyses, in the process of searching, we searched five databases and used the keywords with metformin, glyburide, insulin, GDM, and RCT, to assure more and complete articles being included. 12 primary outcomes and 24 secondary outcomes were detected and analyzed; our meta-analysis is more detailed compared with others. In addition, there were two forms of outcomes, forest plots and funnel plots.

A major strength of this study was the comprehensive coverage of the literature achieved by including the most up-to-date review on the topic and including 41 RCTs in the medical literature and the comparison of three drugs (metformin, glyburide, and insulin) in the treatment of GDM patients. Moreover, it is evident from the bias summary that 2 studies were at high risk of two types of bias.

However, there were several limitations to the meta-analysis that deserve comment. First, some of the outcomes were only included by a few studies, and there have been insufficient power to detect important differences between treatment groups. Second, definitions for GDM and some outcomes (e.g., gestational hypertension, neonatal hypoglycemia, and macrosomia) were either not defined by some studies or the definitions varied between studies. Third, none of these studies evaluated long-term maternal and neonatal outcomes. Moreover, the different gestational ages at enrollment might also result in heterogeneity in gestational weight gain.

## 5. Conclusions

In summary, based on the short-term data available, metformin could be a safe and effective treatment for GDM. However, clinicians should pay attention to the relative lack of long-term offspring data with GDM patients treated with metformin. Compared with insulin, glyburide had a higher increase of neonatal hypoglycemia. The other use of glyburide in pregnancy for GDM women appears to be unclear. Clinicians should weigh in practice the condition of patients when selecting different GDM treatment strategy. Further studies with larger sample sizes are required to confirm the long-term maternal and neonatal outcomes in the metformin-treated GDM patients for the safety of metformin as a universal treatment in GDM patients and to reassess the efficacy and safety of glyburide in the treatment of GDM patients.

## Figures and Tables

**Figure 4:**
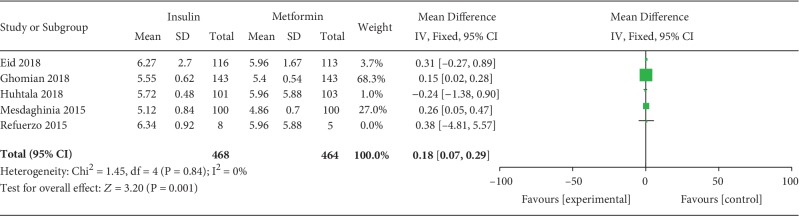
HbA1c% at delivery.

**Figure 5:**
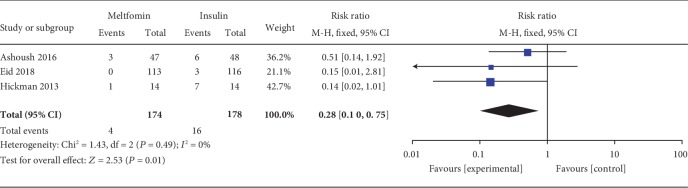
Maternal hypoglycemia.

**Figure 6:**
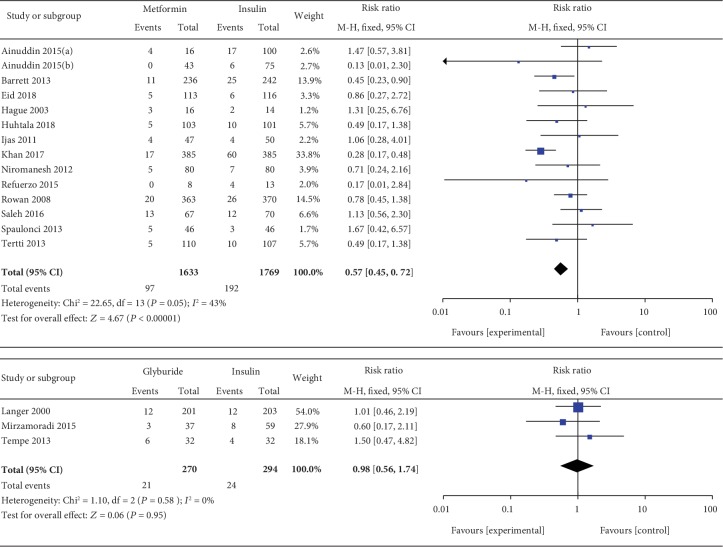
Preeclampsia.

**Figure 7:**
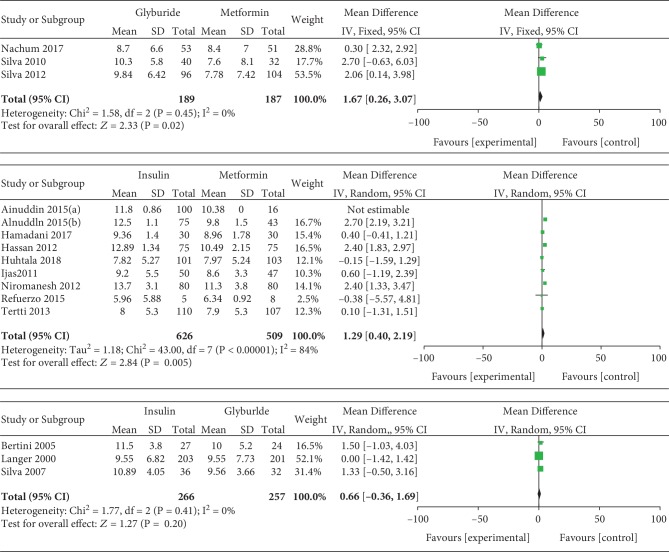
Gestational weight gain.

**Figure 8:**
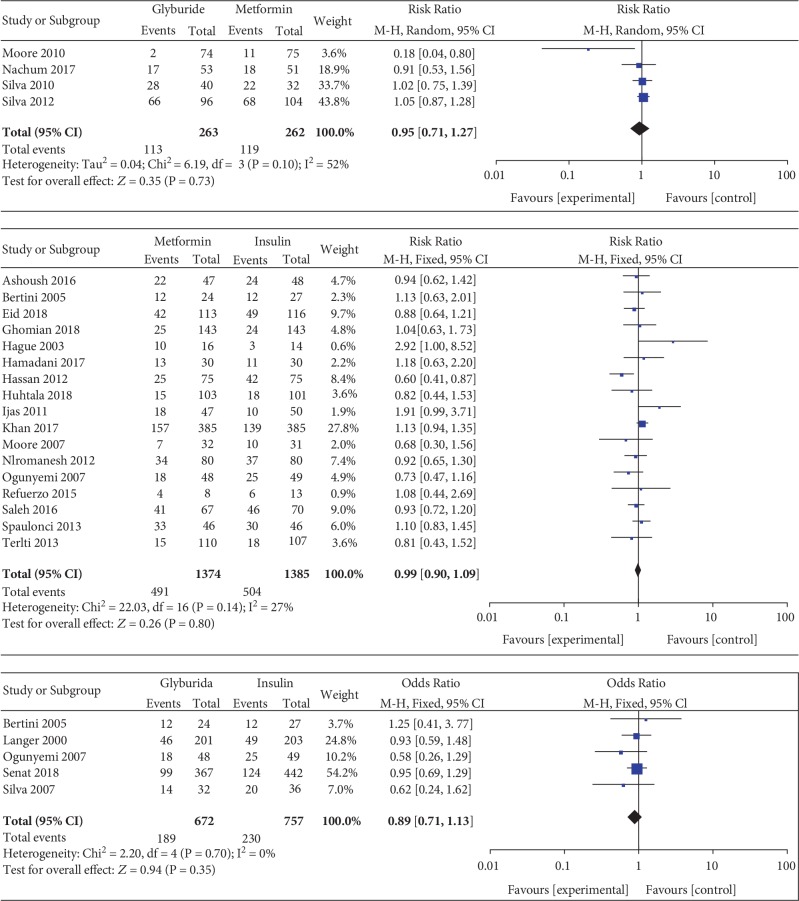
Cesarean section.

**Figure 9:**
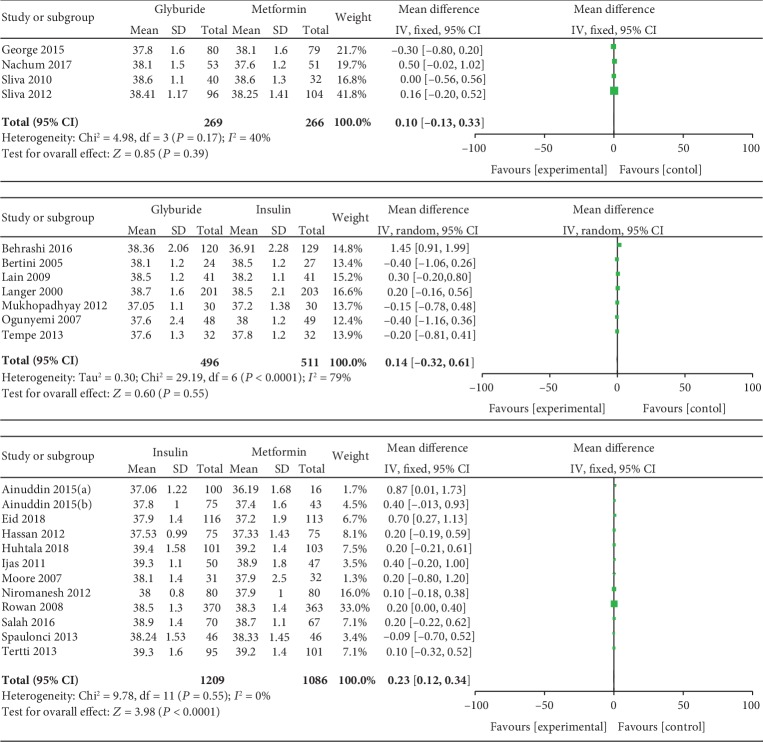
Gestational age at delivery.

**Figure 10:**
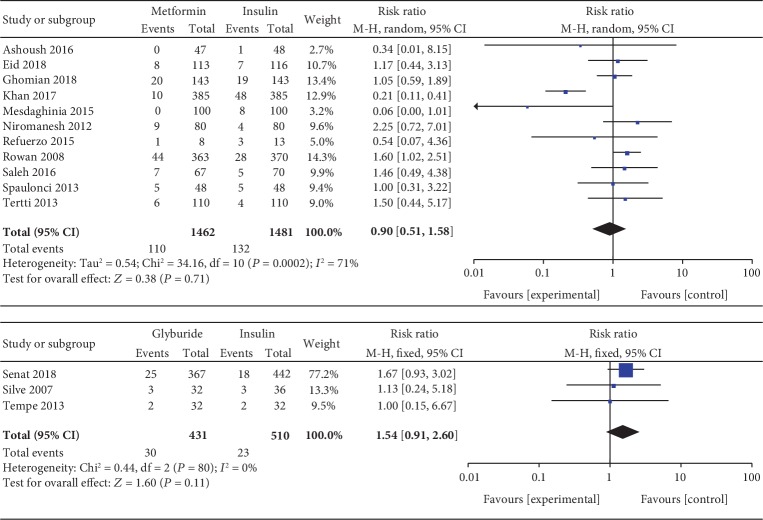
Preterm birth.

**Figure 11:**
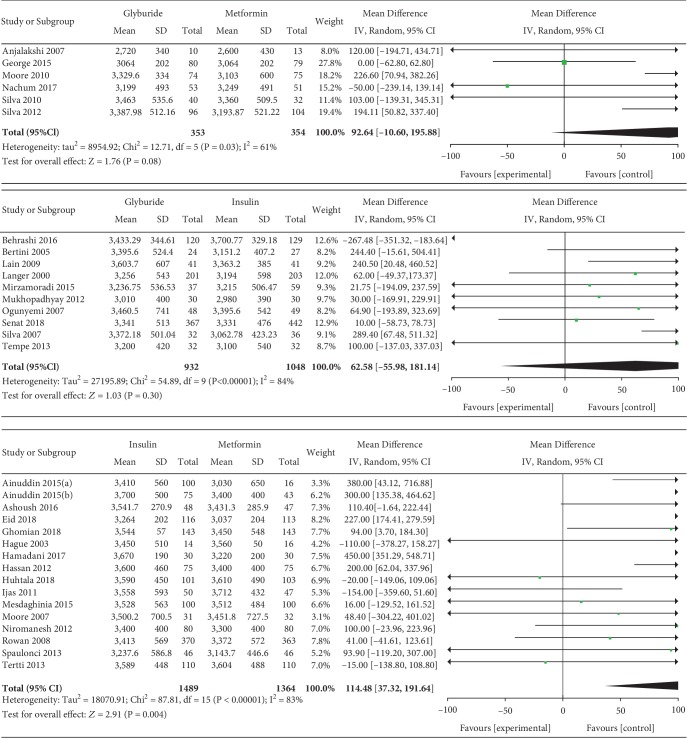
Birth weight.

**Figure 12:**
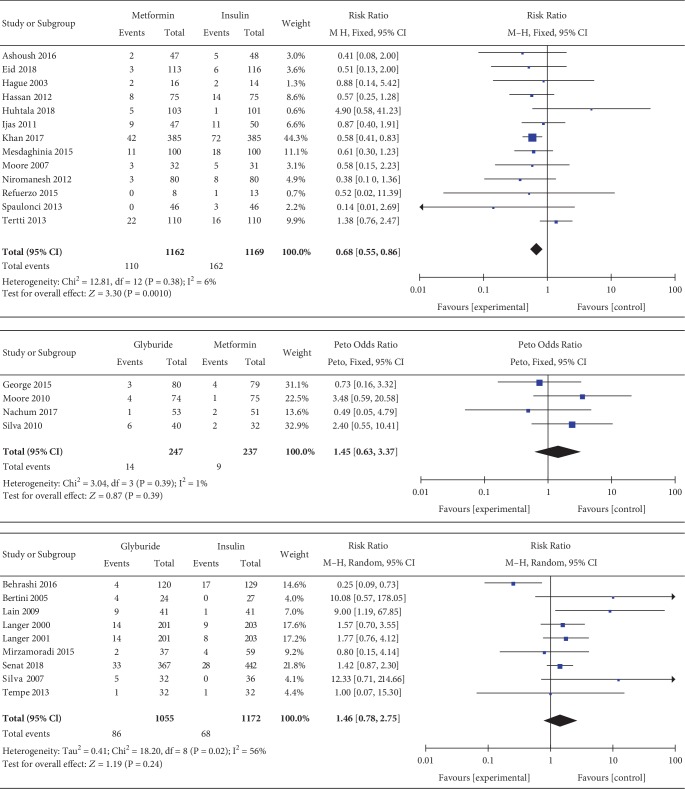
Macrosomia.

**Figure 13:**
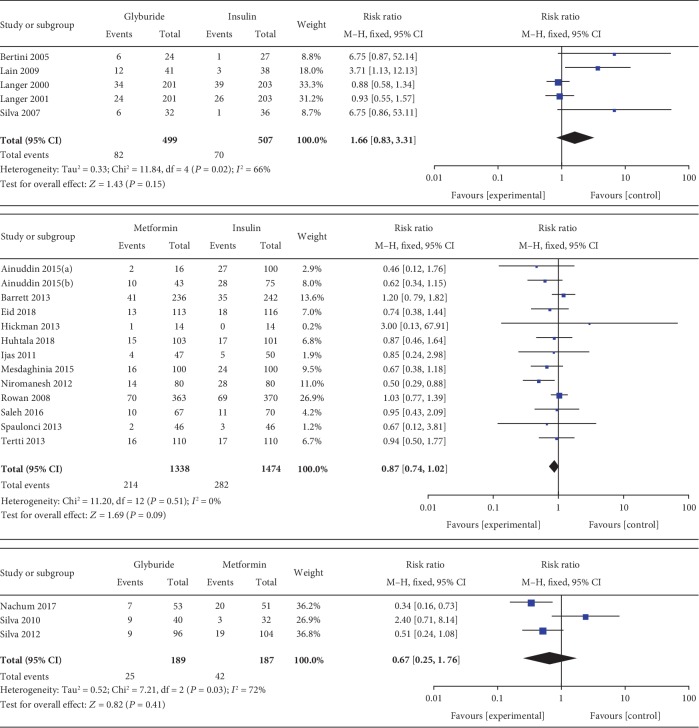
Large for gestational age.

**Figure 14:**
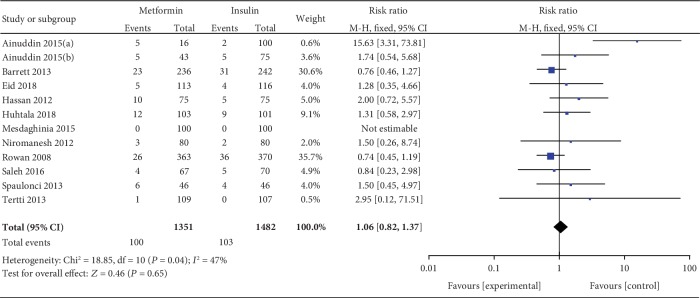
Small for gestational age.

**Figure 15:**
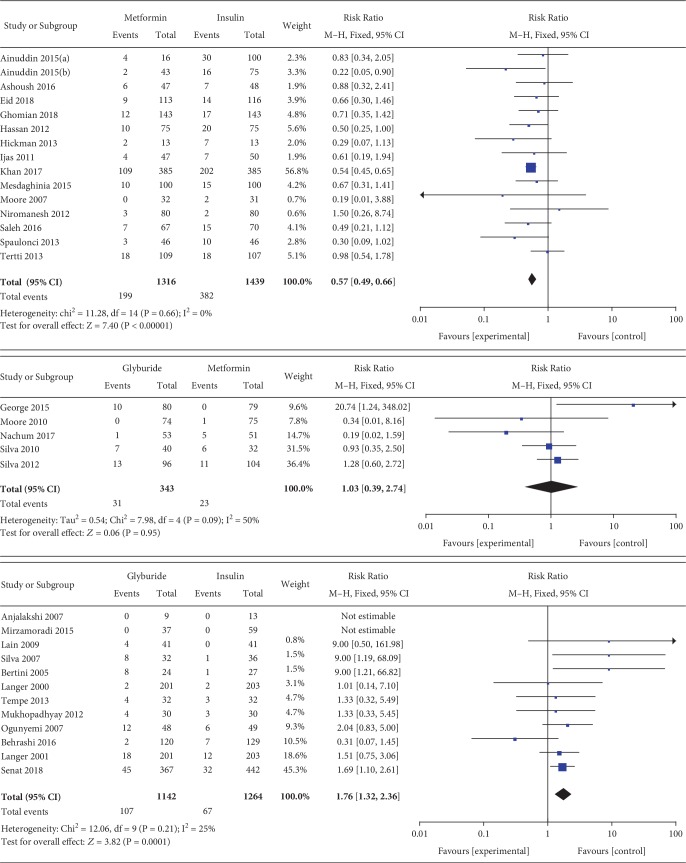
Neonatal hypoglycemia.

**Figure 16:**
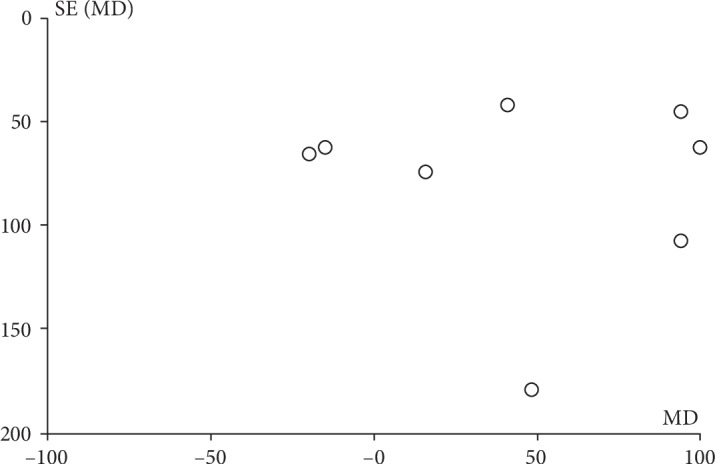
Funnel plots: birth weight (insulin vs. metformin).

**Table 1:** Search strategy.

*Search terms*
Unless otherwise stated, search terms are free text terms; ab = abstract; adj = adjacent; exp = exploded MeSH; MeSH = medical subject heading (Medline medical index term); ot = original title; pt = publication type; sh = MeSH; kw = key word; tw = text word; ti = title; the dollar sign ($) stands for any character(s); the question mark (?) = to substitute for one or no characters.
*PubMed: 2432*
#1 metformin.ti,ab.
#2 melbine.ti,ab.
#3 DMBG.ti,ab.
#4 MET.ti,ab.
#5 dimethylbiguanidium.ti,ab.
#6 dimethyldiguanide.ti,ab.
#7 dimethylguanylguanidine.ti,ab.
#8 glucophage.ti,ab.
#9 glucovance.ti,ab.
#10 #1 OR #2 OR #3 OR #4 OR #5 OR #6 OR #7 OR #8 OR #9 182772
#11 “Metformin”[Mesh] 11462
#12 #10 OR #11 184373
#13 Insulin.ti,ab.
#14 Insulinum.ti,ab.
#15 iletin.ti,ab.
#16 InS.ti,ab.
#17 NPH.ti,ab.
#18 (detemir OR levemir). ti,ab.
#19 (glargin∗ OR lantus). ti,ab.
#20 #13 OR #14 OR #15 OR #16 OR #17 OR #18 OR #19 343653
#21 “Insulin”[Mesh] 177525
#22 #20 OR #21 377267
#23 glyburide.ti,ab.
#24 glibenclamide.ti,ab.
#25 glimepiride.ti,ab.
#26 glipizide.ti,ab.
#27 sulfonylurea.ti,ab.
#28 sulphonylurea.ti,ab.
#29 #23 OR #24 OR #25 OR #26 OR #27 OR #28 15748
#30 “Glyburide”[Mesh] 6051
#31 #29 OR #30 16747
#32pregnan∗.ti,ab.
#33 gestation∗.ti,ab.
#34 GDM.ti,ab.
#35 gestational diabetes.ti,ab.
#36 diabetes mellitus in pregnancy.ti,ab.
#37 (diabetes AND pregnancy).ti,ab.
#38 #32 OR #33 OR #34 OR #35 OR #36 OR #37 573953
#39 “Diabetes, Gestational”[Mesh] 10717
#40 “Pregnancy”[Mesh] 845568
#41 #38 OR #39 OR #40 1001062
#42 (randomized controlled trial[pt] OR controlled clinical trial[pt] OR randomized[ti,ab] OR placebo[ti,ab] OR randomly[ti,ab] OR trial[ti]) OR clinical trials as topic[mesh:noexp] NOT (animals [mh] NOT (humans [mh] AND animals[mh])) 1094992
#43 #12 OR #22 OR #31 566406
#44 #41 AND #43 28852
#45 #44 AND #42 2432
*Embase: 3978*
#1 ‘metformin'/exp 55,211
#2 ‘metformin':ab,ti OR ‘melbine':ab,ti OR ‘dmbg':ab,ti OR ‘met':ab,ti OR ‘dimethylbiguanidium':ab,ti OR ‘dimethyldiguanide':ab,ti OR ‘dimethylguanylguanidine':ab,ti OR ‘glucophage':ab,ti OR ‘glucovance':ab,ti 285,081
#3 #1 OR #2 312,295
#4 ‘insulin'/exp 335,113
#5 ‘insulinum':ab,ti OR ‘insulin':ab,ti OR ‘nph':ab,ti OR ‘detemir':ab,ti OR ‘levemir':ab,ti OR ‘glargin∗':ab,ti OR ‘lantus':ab,ti 460,703
#6 #4 OR #5 543,932
#7 ‘glibenclamide'/exp 23,962
#8 ‘glyburide':ab,ti OR ‘glibenclamide':ab,ti OR ‘glimepiride':ab,ti OR ‘glipizide':ab,ti OR ‘sulfonylurea':ab,ti OR ‘sulphonylurea':ab,ti 23,059
#9 #7 OR #8 34,460
#10 ‘pregnancy'/exp 745,337
#11 ‘pregnancy diabetes mellitus'/exp 32,173
#12 ‘pregnan∗':ab,ti OR ‘gestation∗':ab,ti OR ‘gdm':ab,ti OR ‘gestational diabetes':ab,ti OR ‘diabetes mellitus in pregnancy':ab,ti OR (‘diabetes':ab,ti AND ‘pregnancy':ab,ti) 770,703
#13 #10 OR #11 OR #12 1,057,118
#14 #3 OR #6 OR #9 845,415
#15 #13 AND #14 40,184
#16 #15 AND ([cochrane review]/lim OR [systematic review]/lim OR [meta analysis]/lim OR [controlled clinical trial]/lim OR [randomized controlled trial]/lim) 3978
*Web of Science: 4651*
# 1 Topic: (Metformin) OR Topic: (melbine) OR Topic: (DMBG) OR Topic: (MET) OR Topic: (dimethylbiguanidium) OR Topic: (dimethyldiguanide) OR Topic: (glucophage) OR Topic: (glucovance) 576,285
# 2 Topic: (Insulin) OR Topic: (iletin) OR Topic: (InS) OR Topic: (NPH) OR Topic: (detemir or levemir) OR Topic: (glargin∗ or lantus) 437,814
# 3 Topic: (glyburide) OR Topic: (glibenclamide) OR Topic: (glimepiride) OR Topic: (glipizide) OR Topic: (sulfonylurea) OR Topic: (sulphonylurea) 19,751
# 4 Topic: (Pregnancy) OR Topic: (Diabetes, Gestational) OR Topic: (pregnan∗) OR Topic: (gestation∗) OR Topic: (GDM) OR Topic: (gestational diabetes) OR Topic: (diabetes mellitus in pregnancy) OR Topic: (diabetes AND pregnancy) 488,448
# 5 Topic: (randomized controlled trial) OR Topic: (controlled clinical trial) OR Topic: (randomized) OR Topic: (placebo) OR Topic: (randomly) OR Topic: (trial) OR Topic: (clinical trials as topic) 1,921,062
# 6 #3 OR #2 OR #1 1,027,299
# 7 #4 AND #6 28,082
# 8 #5 AND #7 4,651
*CINAHL: 878*
#1 (MH “Metformin”) 4,429
#2 (AB Metformin OR AB melbine OR AB DMBG OR AB MET OR AB dimethylbiguanidium OR AB dimethyldiguanide OR AB dimethylguanylguanidine OR AB glucophage OR AB glucovance) 55,171
#3 (MH “Insulin+”) 26,217
#4 (MH “Insulin, Short-Acting”) 51
#5 (MH “Insulin, Intermediate-Acting”)20
#6 (MH “Insulin, Long-Acting”)328
#7 (MH “Insulin, Rapid-Acting+”)294
#8 (MH “Protamines”) 181
#9 AB Insulin OR AB Insulinum OR AB iletin OR AB InS OR AB NPH OR AB neutral protamine hegedom OR AB (detemir or levemir) OR AB (glargin∗ or lantus) 37,056
#10 (MH “Glyburide”) 607
#11 (MH “Glimepiride”) 82
#12 (MH “Glipizide”) 138
#13 (MH “Sulfonylurea Compounds+”) 2,146
#14 AB Glyburide OR AB glibenclamide OR AB glimepiride OR AB glipizide OR AB sulfonylurea OR AB sulphonylurea 2,290
#15 (MH “Pregnancy+”) 175,387
#16 (MH “Diabetes Mellitus, Gestational”) 5,377
#17 (MH “Pregnancy in Diabetes+”) 7,026
#18 AB Pregnancy OR AB Diabetes, Gestational OR AB pregnan∗ OR AB gestation∗ OR AB gdm OR AB gestational diabetes OR AB (diabetes and pregnancy) OR AB diabetes mellitus in pregnancy OR AB gestational diabetes OR AB gestational diabetes mellitus 92,542
#19 #1 OR #2 11,271
#20 #3 OR #4 OR #5 OR #6 OR #7 OR #8 OR #9 7,514
#21 #10 OR #11 OR #12 OR #13 OR #14 559
#22 #15 OR #16 OR #17 OR #18 42,697
#23 #19 OR #20 OR #21 18,539
#24 #22 AND #23 878
*Cochrane Library: 8814*
#1 [Metformin] [Mesh] 3299
#2 (Metformin): ti,ab,kw OR (MET):ti,ab,kw 27959
#3 (dimethyldiguanide): ti,ab,kw OR (dimethylguanylguanidine):ti,ab,kw OR (glucophage):ti,ab,kw OR (glucovance):ti,ab,kw (Word variations have been searched) 103
#4 #1 OR #2 OR #3 27963
#5 (Insulin): ti,ab,kw OR (iletin):ti,ab,kw OR (InS):ti,ab,kw 1146182
#6 (NPH): ti,ab,kw OR (neutral protamine hegedom):ti,ab,kw OR (detemir or levemir):ti,ab,kw OR (glargin∗ or lantus):ti,ab,kw 2517
#7 [Insulins] [Mesh] 12622
#8 #5 OR #6 OR #7 1146186
#9 [Glyburide] [Mesh] 568
#10 (glyburide): ti,ab,kw OR (glibenclamide):ti,ab,kw OR (glimepiride):ti,ab,kw OR (glipizide):ti,ab,kw AND (sulfonylurea):ti,ab,kw 1981
#11 (sulphonylurea):ti,ab,kw 545
#12 #9 OR #10 OR #11 2318
#13 #4 OR #8 OR #12 1146617
#14 [Pregnancy] [Mesh] 6925
#15 [Diabetes, Gestational] [Mesh] 692
#16 (diabetes AND pregnancy): ti,ab,kw 2228
#17 (GDM): ti,ab,kw OR (gestational diabetes):ti,ab,kw OR (diabetes mellitus in pregnancy):ti,ab,kw OR (diabetes AND pregnancy):ti,ab,kw 2468
#18 #14 OR #15 OR #16 OR #17 9109
#19 #13 AND #18 7968

**Table 2:** Demographics of included studies.

First author	Year	Location	Groups	No. of subjects enrolled	Age, mean (SD) (y)	BMI at entry into the study, mean (SD) (kg/m^2^)	Gestational age at entry into the study (weeks)	Initiated dose	Maximum dose
Refuerzo	2015	America	Metformin	8	30.9 (5.5)	35.9 (5.2)	Unstated	500 mg/d	2500 mg/d
			Insulin	13	32.3 (4.3)	40.1 (8.4)	Unstated	1-13 wks: 0.7 U/kg/d 14-27 wks: 0.8 U/kg/d ≥28 wks: 0.9-1.0 U/kg/d	Unstated

Anjalakshi	2007	India	Glyburide	10	24.9 (3.73)	22.82 (3.50)	22.5 (4.72)	0.625 mg/wk	Unstated
			Insulin	13	27.46 (5.83)	25.32 (5.14)	22.62 (5.62)	0.1 U/kg/d	Unstated

Huhtala	2018	Finland	Metformin	110	31.9 (5.01)	29.5 (5.91)	Unstated	500 mg/d	2000 mg/d
			Insulin	107	32.0 (5.47)	28.9 (4.71)	Unstated	Unstated	Unstated

Behrashi	2016	Iran	Glyburide	120	30.69 (7.194)	21.94 (2.800)	24.89 (3.90)	1.25 mg/d	20 mg/d
			Insulin	129	29.98 (7.033)	22.59 (3.094)	24.48 (4.51)	0.2 IU/kg/d	Unstated

Mirzamoradi	2015	Iran	Glyburide	37	29.50 (4.06)	30.18 (5.35)	Unstated	1.25 mg/d	20 mg/d
			Insulin	59	31.18 (5.01)	31.77 (5.11)	Unstated	0.4 U/kg/d	Unstated

Langer	2000	America	Glyburide	201	29 (7)	Unstated	24 (7)	2.5 mg/d	20 mg/d
			Insulin	203	30 (6)	Unstated	25 (7)	0.7 U/kg/d	Unstated

Khan	2017	Pakistan	Metformin	385	24.92 (2.57)	22.08 (2.98)	27.94 (2.57)	500 mg/d	Unstated
			Insulin	385	28.01 (2.53)	23.82 (2.81)	29.92 (2.27)	0.7 U/kg/d	Unstated

George	2015	India	Glyburide	80	33.6 (4.6)	28.8 (4.0)	29.7 (3.7)	2.5 mg/d	15 mg/d
			Metformin	79	33.4 (4.4)	28.7 (4.4)	29.3 (3.3)	500 mg/d	2000 mg/d

Mesdaghinia	2013	Iran	Insulin	100	30.2 (5.9)	Unstated	28.9 (3.8)	0.5 IU/kg/d	Unstated
			Metformin	100	29.6 (5.3)	Unstated	27.9 (3.22)	500 mg/d	2000 mg/d

Hague	2003	Australia	Insulin	14	34.1 (3.70)	37.9 (6.87)	30.4 (4.67)	Unstated	Unstated
			Metformin	16	33.7 (4.44)	39.5 (6.94)	29.8 (4.49)	Unstated	Unstated

Sénat	2018	France	Glyburide	367	32.5 (5.1)	30.7 (5.1)	Unstated	2.5 mg/d	20 mg/d
			Insulin	442	32.6 (5.3)	31.1 (5.4)	Unstated	4 IU/d	Unstated

Waheed	2013	Pakistan	Insulin	34	29.82 (4.58)	Unstated	Unstated	Unstated	Unstated
			Metformin	34	29.35 (4.97)	Unstated	Unstated	500 mg/d	1500 mg/d

Reynolds	2017	UK	Glyburide	13	33.0 (5.1)	Unstated	29.6 (6.3)	2.5 mg/d	20 mg/d
			Insulin	10	34.59 (4.9)	Unstated	31.5 (2.2)	Unstated	Unstated

Tempe	2013	India	Glyburide	32	27.5 (3.04)	Unstated	25.9 (5.1)	2.5 mg/d	20 mg/d
			Insulin	32	26.9 (3.06)	Unstated	27.3 (4.1)	4 IU/d	Unstated

Nachum	2017	Israel	Glyburide	53	32.8 (5.0)	28.6 (4.7)	Unstated	2.5 mg/d	20 mg/d
			Metformin	51	33.6 (5.3)	28.6 (5.5)	Unstated	850 mg/d	2550 mg/d

Langer	2001	America	Glyburide	201	Unstated	Unstated	Unstated	2.5 mg/d	20 mg/d
			Insulin	203	Unstated	Unstated	Unstated	0.7 U/kg/d	Unstated

Ashoush	2016	Egypt	Metformin	47	32.1 (3.2)	31.1 (1.3)	28.2 (1.3)	1000 mg/d	2500 mg/d
			Insulin	48	31.6 (2.8)	31.4 (1.5)	27.8 (1.4)	0.7 U/kg/d	Unstated

Eid	2018	Egypt	Metformin	113	31.6 (3.6)	29.44 (4.53)	27.4 (3.9)	500 mg/d	2500 mg/d
			Insulin	116	30.4 (3.5)	30.5 (4.2)	28.1 (3.1)	0.7 U/kg/d	Unstated

Barrett	2013	Australia	Metformin	236	Unstated	Unstated	Unstated	Unstated	Unstated
			Insulin	242	Unstated	Unstated	Unstated	Unstated	Unstated

Moore	2007	America	Insulin	31	27.7 (6.7)	35.3 (6.7)	28.9 (5.0)	0.7 U/kg/d	Unstated
			Metformin	32	27.1 (4.7)	39.7 (9.0)	27.8 (6.5)	1000 mg/d	2000 mg/d

Silva	2010	Brazil	Glyburide	40	31.5 (5.4)	28.8 (5.8)	25.6 (6.4)	2.5 mg/d	20 mg/d
			Metformin	32	33.6 (5.8)	30.3 (5.7)	26.8 (6.0)	1000 mg/d	2500 mg/d

Moore	2010	America	Glyburide	74	29.6 (7.8)	32.7 (7.0)	29.1 (5.0)	5 mg/d	20 mg/d
			Metformin	75	31 (7.1)	32.8 (5.8)	27.3 (6.8)	500 mg/d	2000 mg/d

Niromanesh	2012	Iran	Metformin	80	30.7 (5.5)	28.1 (4.0)	28.7 (3.7)	500 mg/d	2000 mg/d
			Insulin	80	31.8 (5.1)	27.1 (2.1)	28.6 (3.6)	0.7 U/kg/d	Unstated

Hickman	2013	America	Metformin	14	Unstated	Unstated	Unstated	500 mg/d	Unstated
			Insulin	14	Unstated	Unstated	Unstated	0.7 U/kg/d	Unstated

Hassan	2012	Pakistan	Insulin	75	30.88 (3.6)	28.74 (2.69)	29.20 (1.48)	Unstated	Unstated
			Metformin	75	30.29 (3.06)	29.17 (1.94)	29.53 (1.33)	500 mg/d	3000 mg/d

Ijäs	2011	Finland	Metformin	47	32.3 (5.6)	31.5 (6.5)	30 (4.9)	1st week: 750 mg/d 2nd week: 750 mg/d After 3rd week: 750 mg/d	Unstated
			Insulin	50	31.7 (6.1)	30.8 (5.4)	30 (4.0)	Unstated	Unstated

Ainuddin	2015	Pakistan	Metformin	16	31.75 (2.82)	28.25 (1.98)	10.75 (5.98)	500 mg/d	2500 mg/d
			Insulin	100	33.73 (2.95)	32.96 (4.04)	9.57 (5.20)	14-27 wks: 0.7 U/kg 28-32 wks: 0.8 U/kg 32-36 wks: 0.9 U/kg ≥36 wks: 1 U/kg	Unstated

Rowan	2008	New Zealand	Metformin	363	33.5 (5.4)	35.1 (8.3)	30.2 (3.3)	500 mg/d	2500 mg/d
			Insulin	370	33.0 (5.1)	34.6 (7.2)	30.1 (3.2)	Unstated	Unstated

Beyuo	2015	Ghana	Metformin	43	33.51 (4.67)	33.47 (6.95)	28.13 (2.30)	500 mg/d	2500 mg/d
			Insulin	40	33.10 (4.56)	32.61 (6.21)	28.26 (2.46)	0.3 IU/kg/d	Unstated

Hamadani	2017	Pakistan	Metformin	30	30.26 (3.97)	22.94 (5.86)	28.13 (2.30)	500 mg/d	2000 mg/d
			Insulin	30	29.63 (3.81)	23.43 (5.06)	28.26 (2.46)	Unstated	Unstated

Ainuddin	2015	Pakistan	Metformin	43	30.6 (2.9)	Unstated	29.9 (1.1)	500 mg/d	2500 mg/d
			Insulin	75	31 (4)	Unstated	29.2 (1.5)	0.9 U/kg/d	Unstated

Tertti	2013	Finland	Metformin	111	31.9 (5.0)	29.4 (5.9)	30.3 (2.0)	500 mg/d	1000 mg/d
			Insulin	107	32.1 (5.4)	28.9 (4.7)	30.4 (1.8)	Unstated	Unstated

Lain	2009	America	Insulin	41	31.2 (5.9)	30.9 (5.7)	30.6 (2.2)	0.8 U/kg	Unstated
			Glyburide	41	32.2 (5.0)	33.4 ± 12.9	30.8 (2.5)	2.5 mg/d	Unstated

Mukhopadhyay	2012	India	Glyburide	30	26.3 (4.6)	23.7 (2.7)	28.3 (2.2)	2.5 mg/d	20 mg/d
			Insulin	30	26 (4.3)	23 (2.9)	27.4 (2.7)	0.7 U/kg/d	Unstated

Silva	2012	Brazil	Glyburide	96	31.29 (5.36)	28.61 (5.88)	25.44 (7.13)	2.5 mg/d	20 mg/d
			Metformin	104	32.63 (5.61)	28.69 (5.37)	26.96 (6.44)	500 mg/d	2500 mg/d

Spaulonci	2013	Brazil	Metformin	46	31.93 (6.02)	31.97 (4.71)	32.18 (3.70)	1700 mg/d	2550 mg/d
			Insulin	46	32.76 (4.66)	31.31 (5.80)	32.05 (3.50)	0.4 U/kg/d	Unstated

Ghomian	2018	Iran	Metformin	143	28.30 (5.25)	23.73 (1.87)	24.80 (1.45)	500 mg/d	1500 mg/d
			Insulin	143	28.41 (6.36)	24.0 (2.10)	25.10 (1.05)	0.1 IU/kg	Unstated

Saleh	2016	Egypt	Metformin	67	31 (3.42)	30.52 (3.17)	Unstated	500 mg/d	3000 mg/d
			Insulin	70	29.8 (2.18)	31.58 (30.12)	Unstated	1-13 wks: 0.6 U/kg/d 14-27 wks: 0.7 U/kg/d 28-32 wks: 0.8 U/kg/d 32-36 wks: 0.9 U/kg/d ≥36 wks: 1 U/kg/d	Unstated

Refuerzo	2015	America	Metformin	8	30.9 (5.5)	35.9 (5.2)	Unstated	500 mg/d	2500 mg/d
			Insulin	13	32.3 (4.3)	40.1 (8.4)	Unstated	1-13 wks: 0.7 U/kg/d 14-27 wks: 0.8 U/kg/d ≥28 wks: 0.9-1.0 U/kg/d	Unstated

Anjalakshi	2007	India	Glyburide	10	24.9 (3.73)	22.82 (3.50)	22.5 (4.72)	0.625 mg/wk	Unstated
			Insulin	13	27.46 (5.83)	25.32 (5.14)	22.62 (5.62)	0.1 U/kg/d	Unstated

Huhtala	2018	Finland	Metformin	110	31.9 (5.01)	29.5 (5.91)	Unstated	500 mg/d	2000 mg/d
			Insulin	107	32.0 (5.47)	28.9 (4.71)	Unstated	Unstated	Unstated

Behrashi	2016	Iran	Glyburide	120	30.69 (7.194)	21.94 (2.80)	24.89 (3.90)	1.25 mg/d	20 mg/d
			Insulin	129	29.98 (7.033)	22.59 (3.094)	24.48 (4.51)	0.2 IU/kg/d	Unstated

Mirzamoradi	2015	Iran	Glyburide	37	29.50 (4.06)	30.18 (5.35)	Unstated	1.25 mg/d	20 mg/d
			Insulin	59	31.18 (5.01)	31.77 (5.11)	Unstated	0.4 U/kg/d	Unstated

Langer	2000	America	Glyburide	201	29 (7)	Unstated	24 (7)	2.5 mg/d	20 mg/d
			Insulin	203	30 (6)	Unstated	25 (7)	0.7 U/kg/d	Unstated

Khan	2017	Pakistan	Metformin	385	24.92 (2.57)	22.08 (2.98)	27.94 (2.57)	500 mg/d	Unstated
			Insulin	385	28.01 (2.53)	23.82 (2.81)	29.92 (2.27)	0.7 U/kg/d	Unstated

George	2015	India	Glyburide	80	33.6 (4.6)	28.8 (4.0)	29.7 (3.7)	2.5 mg/d	15 mg/d
			Metformin	79	33.4 (4.4)	28.7 (4.4)	29.3 (3.3)	500 mg/d	2000 mg/d

Mesdaghinia	2013	Iran	Insulin	100	30.2 (5.9)	Unstated	28.9 (3.8)	0.5 IU/kg/d	Unstated
			Metformin	100	29.6 (5.3)	Unstated	27.9 (3.22)	500 mg/d	2000 mg/d

Hague	2003	Australia	Insulin	14	34.1 (3.70)	37.9 (6.87)	30.4 (4.6)	Unstated	Unstated
			Metformin	16	33.7 (4.44)	39.5 (6.94)	29.8 (4.49)	Unstated	Unstated

Sénat	2018	France	Glyburide	367	32.5 (5.1)	30.7 (5.1)	Unstated	2.5 mg/d	20 mg/d
			Insulin	442	32.6 (5.3)	31.1 (5.4)	Unstated	4 IU/d	Unstated

Waheed	2013	Pakistan	Insulin	34	29.82 (4.58)	Unstated	Unstated	Unstated	Unstated
			Metformin	34	29.35 (4.97)	Unstated	Unstated	500 mg/d	1500 mg/d

Reynolds	2017	UK	Glyburide	13	33.0 (5.1)	Unstated	29.6 (6.3)	2.5 mg/d	20 mg/d
			Insulin	10	34.5 (4.9)	Unstated	31.5 (2.2)	Unstated	Unstated

Tempe	2013	India	Glyburide	32	27.5 (3.04)	Unstated	25.9 (5.1)	2.5 mg/d	20 mg/d
			Insulin	32	26.9 (3.06)	Unstated	27.3 (4.1)	4 IU/d	Unstated

Nachum	2017	Israel	Glyburide	53	32.8 (5.0)	28.6 (4.7)	Unstated	2.5 mg/d	20 mg/d
			Metformin	51	33.6 (5.3)	28.6 (5.5)	Unstated	850 mg/d	2550 mg/d

Langer	2001	America	Glyburide	201	Unstated	Unstated	Unstated	2.5 mg/d	20 mg/d
			Insulin	203	Unstated	Unstated	Unstated	0.7 U/kg/d	Unstated

Ashoush	2016	Egypt	Metformin	47	32.1 (3.2)	31.1 (1.3)	28.2 (1.3)	1000 mg/d	2500 mg/d
			Insulin	48	31.6 (2.8)	31.4 (1.5)	27.8 (1.4)	0.7 U/kg/day	Unstated

Eid	2018	Egypt	Metformin	113	31.6 (3.6)	29.44 (4.53)	27.4 (3.9)	500 mg/d	2500 mg/d
			Insulin	116	30.4 (3.5)	30.5 (4.2)	28.1 (3.1)	0.7 U/kg/d	Unstated

Barrett	2013	Australia	Metformin	236	Unstated	Unstated	Unstated	Unstated	Unstated
			Insulin	242	Unstated	Unstated	Unstated	Unstated	Unstated

Moore	2007	America	Insulin	31	27.7 (6.7)	35.3 (6.7)	28.9 (5.0)	0.7 U/kg/d	Unstated
			Metformin	32	27.1 (4.7)	39.7 (9.0)	27.8 (6.5)	1000 mg/d	2000 mg/d

Silva	2010	Brazil	Glyburide	40	31.5 (5.4)	28.8 (5.8)	25.6 (6.4)	2.5 mg/d	20 mg/d
			Metformin	32	33.6 (5.8)	30.3 (5.7)	26.8 (6.0)	500 mg/d	2500 mg/d

Moore	2010	America	Glyburide	74	29.6 (7.8)	32.7 (7.0)	29.1 (5.0)	5 mg/d	20 mg/d
			Metformin	75	31 (7.1)	32.8 (5.8)	27.3 (6.8)	500 mg/d	2000 mg/d

Niromanesh	2012	Iran	Metformin	80	30.7 (5.5)	28.1 (4.0)	28.7 (3.7)	500 mg/d	2000 mg/d
			Insulin	80	31.8 (5.1)	27.1 (2.1)	28.6 (3.6)	0.7 U/kg/d	Unstated

Hickman	2013	America	Metformin	14	Unstated	Unstated	Unstated	500 mg/d	Unstated
			Insulin	14	Unstated	Unstated	Unstated	0.7 U/kg/d	Unstated

Hassan	2012	Pakistan	Insulin	75	30.88 (3.6)	28.74 (2.69)	29.20 (1.48)	Unstated	Unstated
			Metformin	75	30.29 (3.06)	29.17 (1.94)	29.53 (1.33)	500 mg/d	3000 mg/d

Ogunyemi	2007	America	Glyburide	48	Unstated	32.0 (7.6)	28.1 (7.6)	Unstated	Unstated
			Insulin	49	Unstated	30.8 (6.9)	24.6 (8.0)	Unstated	Unstated

Bertini	2005	Brazil	Glyburide	24	31.2 (4.5)	27.5 ± 5.8	Unstated	5 mg/d	20 mg/d
			Insulin	27	28.7 (6.0)	27.0 ± 7.2	Unstated	Unstated	Unstated

Silva	2007	Brazil	Glyburide	32	31.62 (4.19)	27.53 ± 5.11	26.62 ± 4.25	Unstated	Unstated
			Insulin	36	29.94 (6.02)	27.94 ± 6.81	25.61 ± 5.87	Unstated	Unstated

IRB: institutional review board; UT Health: University of Texas Health Science Center; WHO: World Health Organization; ADIPS: Australasian Diabetes in Pregnancy Society; IADPSG: International Association of the Diabetes and Pregnancy Study Groups; ADA: American Diabetes Association.

## Supplementary Materials

Supplementary Materials According to the suggestion, when the number of events was too low (*P* < 0.05), Peto odds ratio was used to analyze the outcome index. As demonstrated, these ten outcome indexes included macrosomia (glyburide vs. metformin), NICU admission (glyburide vs. insulin), respiratory distress syndrome (RDS) (glyburide vs. insulin), birth injury (metformin vs. insulin), 5-minute Apgar score < 7 (metformin vs. insulin), congenital anomaly (metformin vs. insulin), neonatal hypocalcemia (glyburide vs. insulin), sepsis (metformin vs. insulin), shoulder dystocia (metformin vs. insulin), and transient tachypnea (metformin vs. insulin). As we can see, in the outcome indexes, all of them did not show a statistically significant difference.
