# Supplementary material for: Comparative Efficacy and Safety of Metformin, Glyburide, and Insulin in Treating Gestational Diabetes Mellitus: A Meta-Analysis
Source: J Diabetes Res. 2019 Nov 4;2019:9804708. doi: 10.1155/2019/9804708 (PMC6875019; doi:10.1155/2019/9804708)

1. Macrosomia


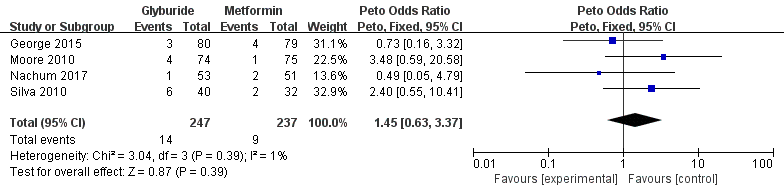


1. NICU admission


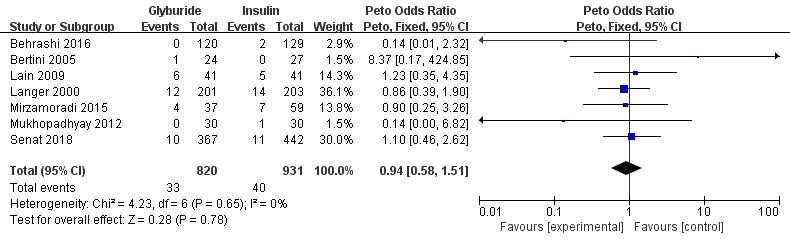


1. Respiratory distress syndrome (RDS)


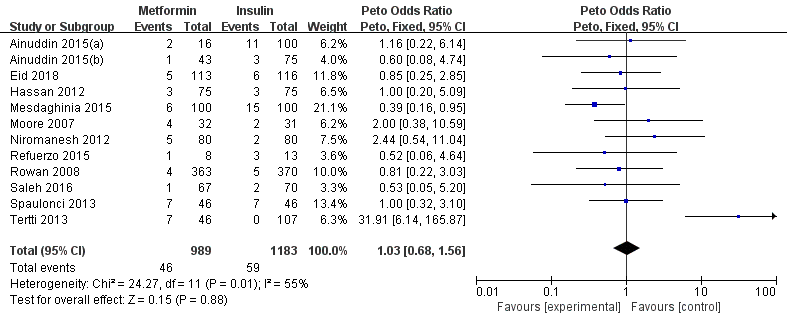


1. Birth injury


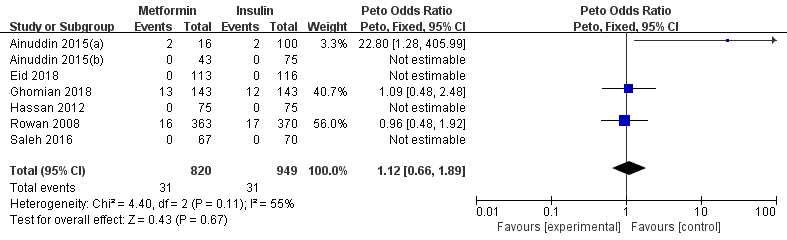


1. 5-min Apgar score < 7


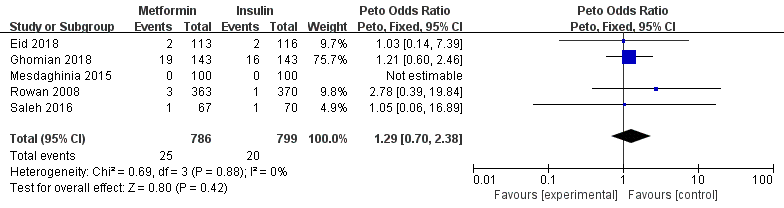


1. Congenial anomaly


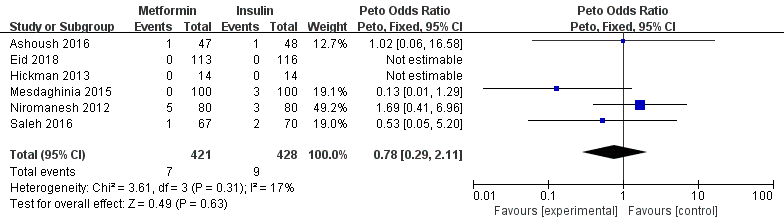


1. Neonatal hypocalcemia


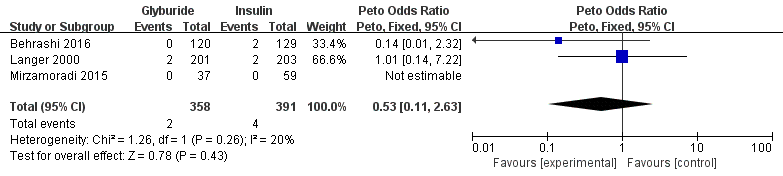


1. Sepsis


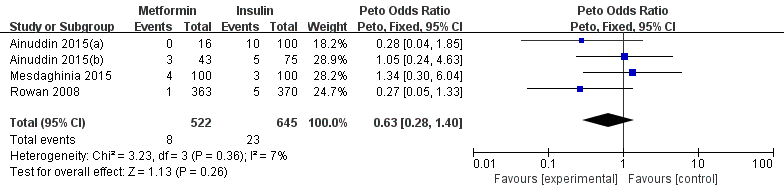


1. Shoulder dystocia


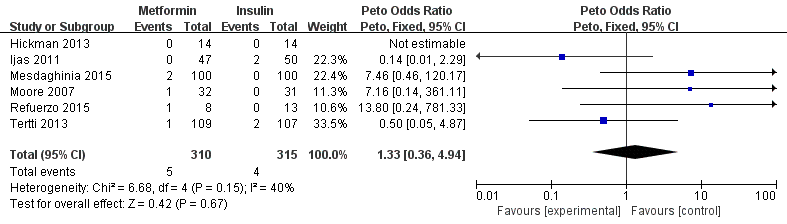


1. Transient tachypnea


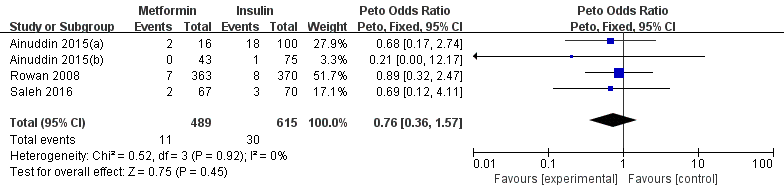

Supplement: Supplementary Materials — According to the suggestion, when the number of events was too low (P < 0.05), Peto odds ratio was used to analyze the outcome index. As demonstrated, these ten outcome indexes included macrosomia (glyburide vs. metformin), NICU admission (glyburide vs. insulin), respiratory distress syndrome (RDS) (glyburide vs. insulin), birth injury (metformin vs. insulin), 5-minute Apgar score < 7 (metformin vs. insulin), congenital anomaly (metformin vs. insulin), neonatal hypocalcemia (glyburide vs. insulin), sepsis (metformin vs. insulin), shoulder dystocia (metformin vs. insulin), and transient tachypnea (metformin vs. insulin). As we can see, in the outcome indexes, all of them did not show a statistically significant difference. [file 9804708.f1.docx]
